# Subcellular location of L1 retrotransposon-encoded ORF1p, reverse transcription products, and DNA sensors in lupus granulocytes

**DOI:** 10.1186/s13100-024-00324-x

**Published:** 2024-06-27

**Authors:** Fatemeh Moadab, Sepideh Sohrabi, Xiaoxing Wang, Rayan Najjar, Justina C. Wolters, Hua Jiang, Wenyan Miao, Donna Romero, Dennis M. Zaller, Megan Tran, Alison Bays, Martin S. Taylor, Rosana Kapeller, John LaCava, Tomas Mustelin

**Affiliations:** 1 Division of Rheumatology, Department of Medicine, University of Washington, Seattle, WA USA; 2 Department of Pediatrics, University Medical Center Groningen, University of Groningen, Groningen, The Netherlands; 3 Laboratory of Cellular and Structural Biology, The Rockefeller University, New York, NY USA; 4 ROME Therapeutics, Boston, MA USA; 5 Department of Pathology, Massachusetts General Hospital, Harvard Medical School, Boston, MA USA; 6 European Research Institute for the Biology of Ageing, University Medical Center Groningen, Groningen, The Netherlands; 7 University of Washington, 750 Republican Street, Room E507, Seattle, WA 98109 USA

**Keywords:** Systemic lupus erythematosus, L1, Retrotransposon, MOV10, ZBP1, CGAS, Z-DNA, Autoantibodies, Neutrophils

## Abstract

**Background:**

Systemic lupus erythematosus (SLE) is a chronic autoimmune disease with an unpredictable course of recurrent exacerbations alternating with more stable disease. SLE is characterized by broad immune activation and autoantibodies against double-stranded DNA and numerous proteins that exist in cells as aggregates with nucleic acids, such as Ro60, MOV10, and the L1 retrotransposon-encoded ORF1p.

**Results:**

Here we report that these 3 proteins are co-expressed and co-localized in a subset of SLE granulocytes and are concentrated in cytosolic dots that also contain DNA: RNA heteroduplexes and the DNA sensor ZBP1, but not cGAS. The DNA: RNA heteroduplexes vanished from the neutrophils when they were treated with a selective inhibitor of the L1 reverse transcriptase. We also report that ORF1p granules escape neutrophils during the extrusion of neutrophil extracellular traps (NETs) and, to a lesser degree, from neutrophils dying by pyroptosis, but not apoptosis.

**Conclusions:**

These results bring new insights into the composition of ORF1p granules in SLE neutrophils and may explain, in part, why proteins in these granules become targeted by autoantibodies in this disease.

## Background

Type I interferons (IFN-Is) play a central role in anti-viral immunity [1]. They are also elevated in a number of diseases, such as systemic lupus erythematosus (SLE) [2–5], dermatomyositis [6], and Sjögren’s syndrome [7], where they accompany autoimmunity against nucleic acids and associated proteins. The cellular sources of these nucleic acids and how they provoke autoimmunity are still incompletely understood [8].

A number of cytosolic DNA and RNA sensors have been discovered in the past decade [9, 10]. They are broadly expressed and play key roles in the cell-intrinsic defense against viral infection, where they detect non-self (e.g., viral) DNA or RNA and relay this information to the innate and adaptive immune systems through the production of IFN-Is, particularly interferon β (IFNβ), and the upregulation of major histocompatibility complex (MHC) molecules, co-stimulatory receptors, and other host defense mechanisms. Recent findings reveal that these nucleic acid sensors are activated also in SLE [11, 12], as well as in Aicardi-Goutières syndrome, a genetic disorder characterized by constitutive IFN-I production [13, 14]. The identities of the endogenous nucleic acid species that trigger the sensors in SLE remain speculative, but may include mitochondrial DNA, unedited double-stranded (ds) RNAs, or RNA: DNA heteroduplexes produced by reverse transcription [8]. Of the three types of reverse transcriptases encoded by the human genome, namely telomerase (encoded by *TERT*), endogenous retroviral *pol*, and retrotransposon reverse transcriptase, the two first are unlikely culprits in SLE: telomerase [15] only extends the protein-covered ends of chromosomes using a template RNA (*TERC*) and cannot produce free DNA. Reverse transcription by retroviral Pol occurs upon cell entry of exogenous retroviruses; none of the endogenous retroviruses in our genome have remained infectious [16], in part because they have lost their Pol activity through inactivating mutations. However, there are reports of active reverse transcriptases encoded by HERV-K10 [17, 18], K11, and the insertionally polymorphic HERV-K113 [19], K115 [20], and a recently reported provirus on Xq21.33 [21]. We do not detect transcripts from any of these loci in neutrophils [22, 23].

On the other hand, retrotransposon-encoded reverse transcriptases abound: over half a million full or partial copies of the retrotransposon referred to as the ‘long interspersed nuclear element-1’ (LINE-1 or L1) exist in our genome, constituting approximately 17% of it [24–27]. While the vast majority of L1 copies are truncated and/or mutated, 80–100 copies of the human-specific L1Hs subfamily are potentially retrotransposition-competent [28] and encode for two proteins, termed ORF1p and ORF2p, the latter having an N-terminal endonuclease domain and a centrally located active reverse transcriptase catalytic domain. Numerous L1Hs loci are expressed in patients with SLE [29, 30, 31], 7 of them full-length [31], but only 3 with an intact reverse transcriptase encoded by *ORF2* [32, 33], which during the canonical retrotransposition life-cycle of L1 reverse transcribes the L1 mRNA to create a new genomic copy if it. In addition, ORF2p can be catalytically active in the cytosol and create DNA: RNA heteroduplexes (which can give rise to ssDNA or dsDNA), even in the absence of ORF1p [34, 35], with its own mRNA or other cellular RNAs, such as Alu transcripts, as templates [36, 37]. This triggers IFNβ production through cGAS activation during cellular senescence [38] and, we hypothesize, in SLE.

Our work on L1 in SLE started with the observation that adult [39] and pediatric [40] SLE patients have autoantibodies of IgG class against ORF1p. The pediatric patients also have anti-ORF1p of IgA class [40]. These autoantibody levels were higher in patients with active disease and declined after effective therapy. They also correlated positively with IFN-I-induced gene expression, several other autoantibodies, and with complement consumption [39, 40]. Anti-ORF1p titers also correlated closely with circulating DNA in complex with neutrophil elastase or myeloperoxidase, suggesting that neutrophil death may be involved in the generation of anti-ORF1p antibodies. Indeed, ORF1p protein was detectable in neutrophils and low-density granulocytes by flow cytometry in patients with active disease [31, 40], but much less in other immune cell lineages. The percentage of ORF1p^+^ cells was low (0–5%) in patients with inactive disease, somewhat higher in patients with moderate disease (SLEDAI < 6; 2–20%) and considerably higher in patients with high disease activity (up to 80%). In the present study, we use mass spectrometry quantitation and immunofluorescence microscopy to study the subcellular location of ORF1p in SLE neutrophils and find that it is concentrated in a number of discrete perinuclear dots, which also contain Ro60, MOV10, as well as DNA: RNA heteroduplexes and the nucleic acid sensor ZBP1. We also document the escape of these ribonuclear particles during neutrophil death.

## Results

### Quantitation of ORF1p in neutrophils from SLE patients and healthy controls

**Fig. 1:**
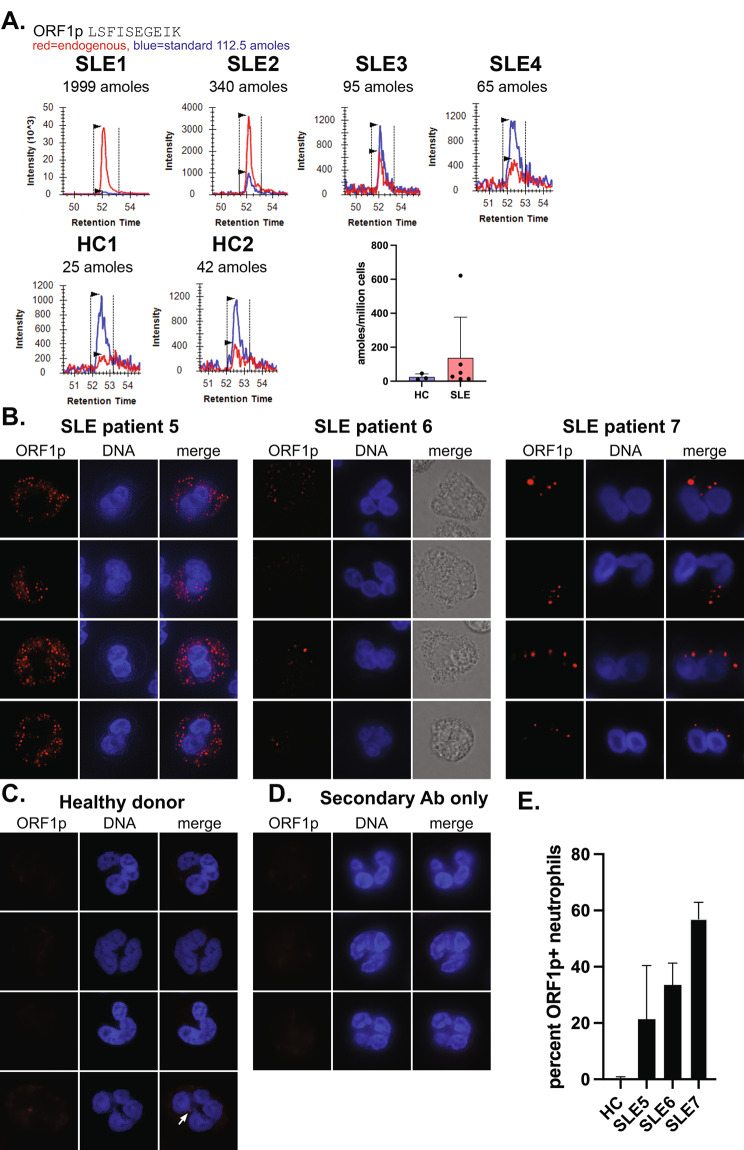
Quantitation and subcellular location of ORF1p in SLE neutrophils. **A.**, Quantitation of ORF1p by targeted mass spectrometry in neutrophils from four different SLE patients and two healthy controls. The amount of ORF1p peptide detected is indicated. Last panel summarizes data from SLE (*n* = 6) and HC (*n* = 3) neutrophils calculated as attomoles per million cells. **B.**, Immunofluorescence staining for ORF1p in four representative neutrophils from three SLE patients with active disease. **C.**, ORF1p in four representative neutrophils from a healthy donor, including the only neutrophil detected with a solitary ORF1p granule (white arrow in bottom panel). **D.**, SLE neutrophils stained with fluorophore-conjugated secondary antibody alone. **E.**, Percent ORF1p^+^ neutrophils in the healthy donor in C and the 3 SLE patients in B expressed as mean and standard deviation from 4 separate 40X views of 60–100 neutrophils each. All shown images were taken with 100 X magnification

ORF1p is detectable by flow cytometry in a subset of neutrophils and low-density granulocytes from SLE patients [31]. Because the accuracy of flow cytometry for low-abundance proteins is not the best, we first quantitated ORF1p in neutrophils from SLE patients and healthy controls (HC) using an independent approach, namely a targeted mass spectrometry assay. This assay compares a known quantity (112.5 amoles) of an isotopically labeled peptide, LSFISEGEIK, with the same peptide derived from ORF1p in the trypsinized sample. By this assay, neutrophils from 6 SLE patients contained 20–622 amoles/10^6^ cells, while HC neutrophils contained 10–20 amoles/10^6^ cells (Fig. 1A). If ORF1p was equally distributed among the neutrophils, this would correspond to up to 375 copies of ORF1p per cell in SLE and 6 copies in healthy donor neutrophils. However, flow cytometry suggests that ORF1p is present only in a subset of SLE neutrophils [40].

### ORF1p is located in cytosolic dots in SLE neutrophils

To learn how ORF1p is distributed among the neutrophils from SLE patients, we visualized it in freshly isolated neutrophils by immunofluorescence microscopy using the well-validated 4H1 monoclonal anti-ORF1p antibody [41] either directly labeled with a fluorophore or by indirect staining by a labeled secondary antibody to amplify the signal. Because neutrophils express Fc receptors and are notorious for background autofluorescence, these experiments were performed in the presence of unlabeled Fc receptor blocking antibodies and an excess of unlabeled mouse serum IgG and exposure times were kept relatively short.

As reported before in cancer cells [42], ORF1p was detectable as concentrated dots throughout the cytosol (Fig. 1B). The percentage of ORF1p^+^ neutrophils and the number of bright dots in them varied between patients: patients with high SLE disease activity index (SLEDAI) scores had a high percentage of ORF1p^+^ neutrophils and mostly 5–10 dots per cells (Fig. 1B), while patients with SLEDAI < 4 had more negative neutrophils (i.e. a lower percent ORF1p^+^ neutrophils) and 1–5 dots in their ORF1p^+^ neutrophils (Fig. 1B). However, while the percentage of ORF1p^+^ neutrophils correlated with disease activity, the number of dots per cell did not. For example, patient SLE5 in Fig. 1B had 20% ORF1p^+^ neutrophils with > 10 dots per cells, while another patient with 100% ORF1p^+^ neutrophils had less than 5 dots per cell. Neutrophils from healthy donors were uniformly ORF1p^–^ with the exception of occasional (< 1%) cells with a faint single dot of ORF1p (indicated by a white arrow in the bottom panel in Fig. 1C). All these microscopy results match the mass spectrometry quantitation (Fig. 1A) and our previous flow cytometry [31, 40].

### ORF1p granules also contain Ro60 and MOV10

**Fig. 2:**
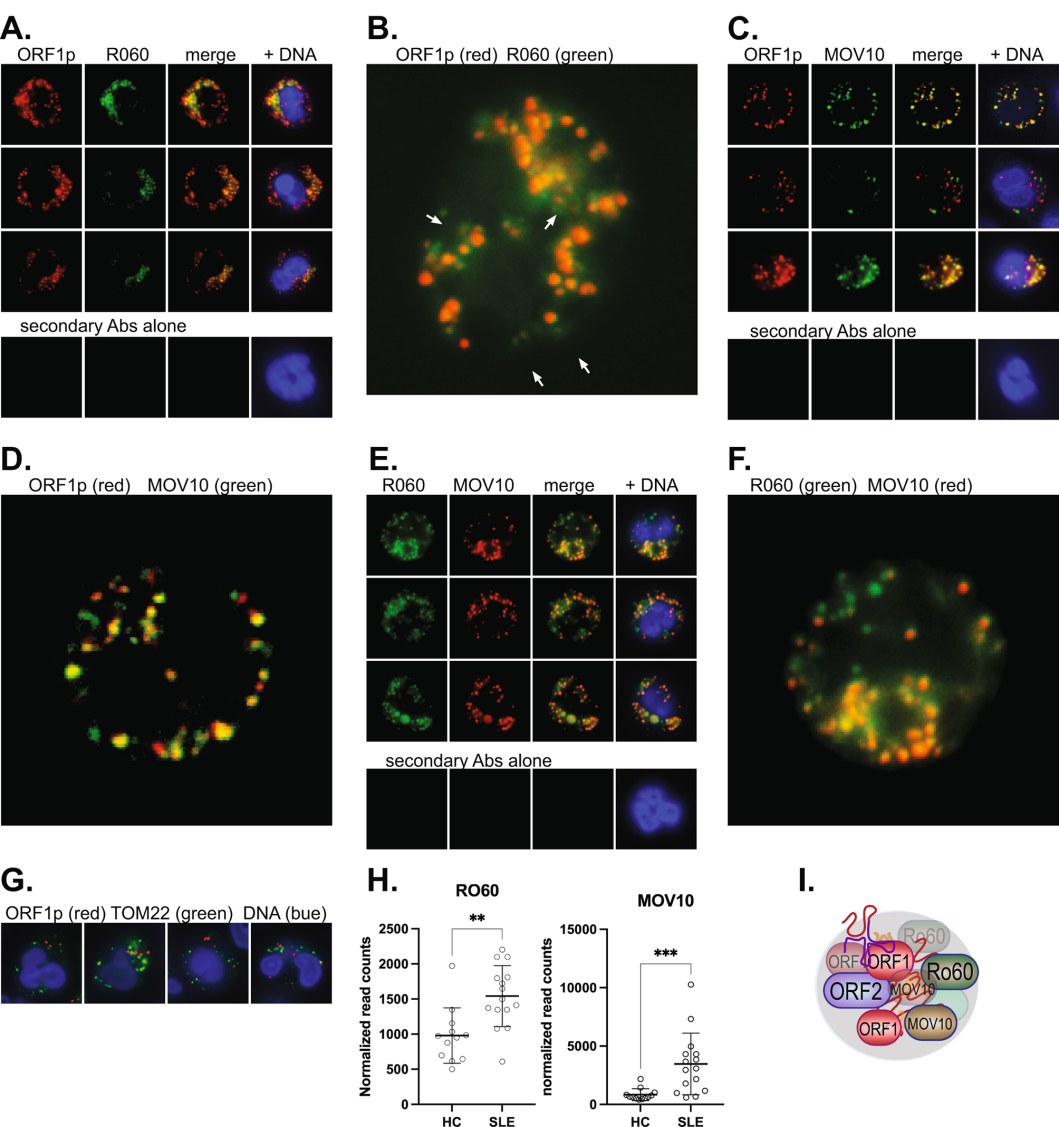
ORF1p is located in cytoplasmic granules largely co-localized with two proteins known to interact with ORF1p in cancer cells, Ro60 and MOV10. **A.**, Three representative neutrophils from a patient with active disease stained with anti-ORF1p (red) and Ro60 (green). The third panel is both colors and the fourth adds DNA stain (blue). Bottom panels show a parallel experiment with fluorophore-conjugated secondary antibody alone. **B.**, Enlarged image of a patient neutrophil to better show the few Ro60 dots that lack ORF1p (white arrows). **C.**, Staining of SLE neutrophils for ORF1p and MOV10. Bottom panels show a patient neutrophil stained with fluorophore-conjugated secondary antibody alone. **D.**, Enlarged image of the patient neutrophil in the top row to better show the mixture of dots with ORF1p, MOV10, or both. **E.**, Similar staining for Ro60 (green) and MOV10 (red). Bottom panels show a patient neutrophil stained with fluorophore-conjugated secondary antibody alone. **F.**, Enlarged image of the patient neutrophil in the top row to better show dots with Ro60, MOV10, or both. **G.**, Control staining for ORF1p (red) and the mitochondrial marker TOM22 (green). All images are with 100 X magnification. **H.**, Expression of R060 and MOV10 in SLE neutrophils (*n* = 15) and HC (*n* = 12) by RNA sequencing. **I.**, Schematic illustration of ORF1p granules also containing R060, MOV10, and many RNA species

When SLE neutrophils were stained for ORF1p together with antibodies against Ro60, it was apparent that the majority of granules containing ORF1p also contained Ro60 (Fig. 2A). Less than 15% of the Ro60 stained dots did not contain ORF1p, while every dot of ORF1p also had Ro60 present (Fig. 2B). It was also clear that those neutrophils that contained ORF1p also expressed Ro60. Secondary antibodies alone did not result in any staining.

Next SLE neutrophils were stained for ORF1p together with an antibody against MOV10, which is also reported to associate with ORF1p in non-hematopoietic cells [43]. These experiments (Fig. 2C) showed that MOV10 was present in most ORF1p granules, but there were also some spots with either protein alone (Fig. 2D). MOV10 was expressed in most cells containing ORF1p and Ro60, but there were a small number of cells that contained ORF1p and Ro60, but not MOV10. Secondary antibodies alone did not stain anything.

Staining of SLE neutrophils with the combination of antibodies against Ro60 and MOV10 confirmed that these two proteins co-localized to a high degree with only a few dots containing Ro60, but not MOV10 (Fig. 2E **and F**). SLE neutrophils stained for only one of these proteins alone gave a very similar dotted pattern of staining (not shown) and omission of the primary antibodies resulted in loss of any staining (lower panels). As an additional control, we stained SLE neutrophils for ORF1p and the mitochondrial protein TOM22, which were not at all colocalized (Fig. 2G).

Healthy donor neutrophils stained with these antibodies showed much less of both Ro60 and MOV10, with only ~ 5% of neutrophils containing them (but no ORF1p). Indeed, the expression levels of the transcripts encoding these two proteins in our RNA sequencing data set (SLE *n* = 15; HC *n* = 12) confirmed that they are expressed at statistically significantly higher levels in SLE neutrophils compared to healthy neutrophils (Fig. 2H).

### DNA: RNA heteroduplexes co-localize with ORF1p in SLE neutrophils

**Fig. 3:**
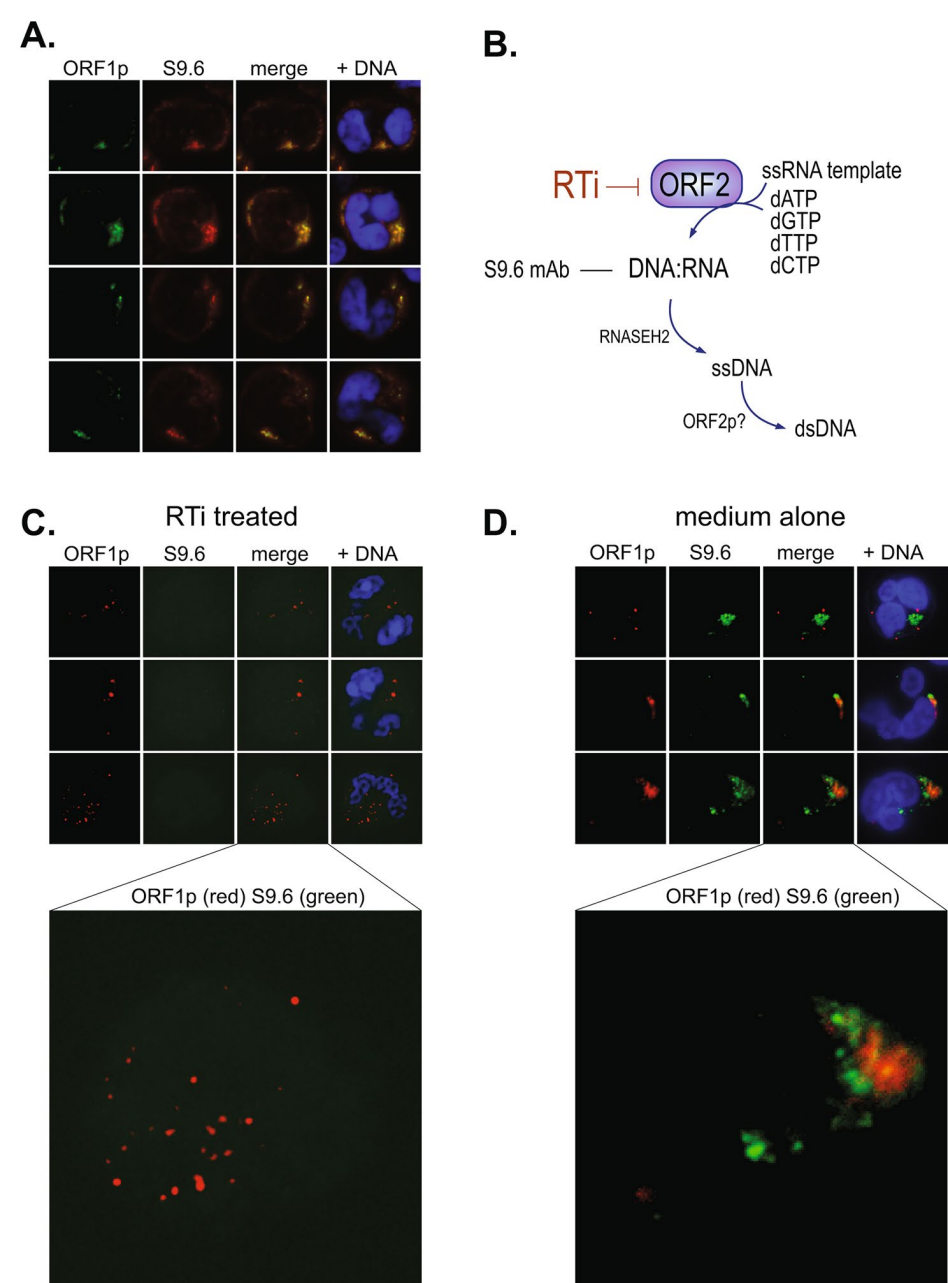
Presence of reverse transcription products in SLE neutrophils. **A.**, Four representative SLE neutrophils stained for ORF1p (green) and RNA: DNA heteroduplexes with the S9.6 mAb (red). **B.**, Schematic illustration of reverse transcription by L1 ORF2p resulting in DNA: RNA recognized by the S9.6 mAb. **C.**, Three SLE neutrophils treated for 4 h with 1 µM of RPT-A and then stained for ORF1p (red) and DNA: RNA by S9.6 (green). The neutrophil in the bottom row is enlarged to better show the absence of RNA: DNA heteroduplexes (green). **D.**, Three SLE neutrophils treated for 4 h with medium alone and then stained for ORF1p (red) and DNA: RNA by S9.6 (green). The neutrophil in the bottom row is enlarged to better show the presence of RNA: DNA heteroduplexes (green). All images are with 100 X magnification

Because the 6-kb bicistronic L1 transcript encodes for both ORF1p and ORF2p, but with the latter translated at a much lower frequency making it near-impossible to detect [44], we decided to visualize its DNA: RNA heteroduplex product using the well-validated S9.6 mAb [45]. This antibody gave a relatively weak, but specific, staining that colocalized to a large extent with ORF1p (Fig. 3A). The staining was similar also in the absence of ORF1p antibodies, excluding the possibility of artifactual cross-reactivity. In contrast, healthy donor neutrophils were completely negative.

To validate that the fluorescence observed with the S9.6 mAb truly represents DNA: RNA heteroduplexes produced by ORF2p-mediated reverse transcription, as illustrated schematically in Fig. 3B, we treated SLE neutrophils with (Fig. 3C) or without (Fig. 3D) 1 µM of a potent ORF2p-selective reverse transcriptase inhibitor RPT-A (see Methods) at 37 °C for 4 h and then fixed and stained them with S9.6 and anti-ORF1p. While ORF1p visualization, cell viability, and morphology remained unchanged, the S9.6 fluorescence was reduced to background. The cells cultured in parallel without inhibitor still contained DNA: RNA heteroduplexes (Fig. 3D). This demonstrates that the signal indeed represented DNA: RNA heteroduplexes produced by ongoing reverse transcription.

### The DNA sensors cGAS and ZBP1 are present in SLE neutrophils

**Fig. 4:**
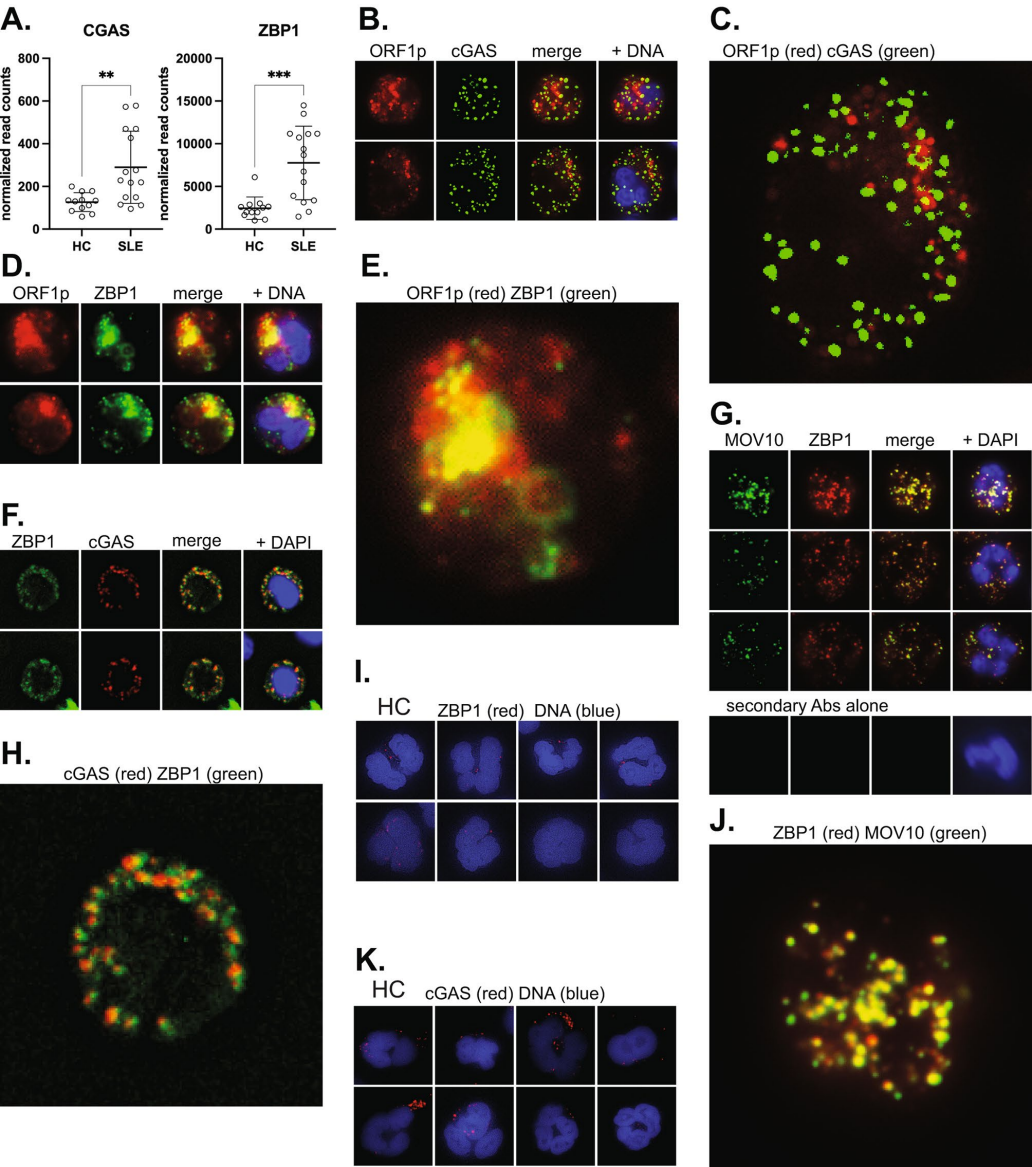
The DNA sensors cGAS and ZBP1 in SLE neutrophils. **A.**, Expression of cGAS and ZBP1 in healthy (*n* = 12) and SLE (*n* = 15) neutrophils by RNA sequencing. **B.**, Staining of two SLE neutrophils for ORF1p (red) and cGAS (green). **C.**, Enlarged view of the bottom neutrophil in B to better visualize the lack of overlap between ORF1p and cGAS. **D.**, Staining of two SLE neutrophils for ORF1p (red) and ZBP1 (green). **E.**, Enlarged view of the last image in D to better visualize the overlap between ORF1p and ZBP1. **F.**, ZBP1 and cGAS in SLE neutrophils. **G.**, MOV10 and ZBP1. Last panels are staining with secondary antibody alone. **H.**, Enlarged view of the last image in F to better visualize the partial co-localization of ORF1p and ZBP1. **I.**, Eight representative healthy control neutrophils stained for ZBP1. **J.**, Enlarged view of the top row neutrophil in G to visualize the co-localization of MOV10 and ZBP1. **K.**, Eight healthy control neutrophils stained for cGAS. All images are with 100 X magnification

Since the ORF2p reverse transcriptase products can trigger DNA sensors that signal to IFNβ production during cellular senescence [38], we assessed the expression of the cytosolic DNA sensor cGAS and nucleic acids sensor ZBP1 in SLE neutrophils. As shown in Fig. 4A, both are expressed at low levels in healthy donor neutrophils, but at statistically significantly higher levels in SLE neutrophils.

In SLE neutrophils, cGAS was present as discrete dots, but they did not overlap with the ORF1p granules (Fig. 4B and **C**). Instead, ZBP1 was to a significant extent co-localized with ORF1p (Fig. 4D and **E**). In agreement with this, cGAS and ZBP1 were mostly in separate locations, but with some overlap (Fig. 4F **and H**), and often closely adjacent to each other. Interestingly, MOV10 was also very closely co-localized with ZBP1 (Fig. 4G **and J**). In healthy donor neutrophils, both cGAS and ZBP1 were present, but particularly the latter in very low quantities and only in a subset of the neutrophils (Fig. 4I **and K**), as also suggested by their lower transcript levels compared to SLE (Fig. 4A).

These data do not prove that ZBP1 is the nucleic acid sensor that detects L1 ORF2p products in SLE neutrophils, but it seems more likely than cGAS because it co-localizes with ORF1p granules that also contain Ro60, MOV10, and DNA: RNA heteroduplexes made by ORF2p. We cannot, of course, exclude the possibility that cGAS participates in sensing DNA: RNA duplexes, or the dsDNA that results from a second round of reverse transcription. ZBP1, on the other hand, may recognize these nucleic acid species, particularly if they adopt the Z-configuration.

### Externalization of ORF1p granules during programmed cell death of SLE neutrophils

**Fig. 5:**
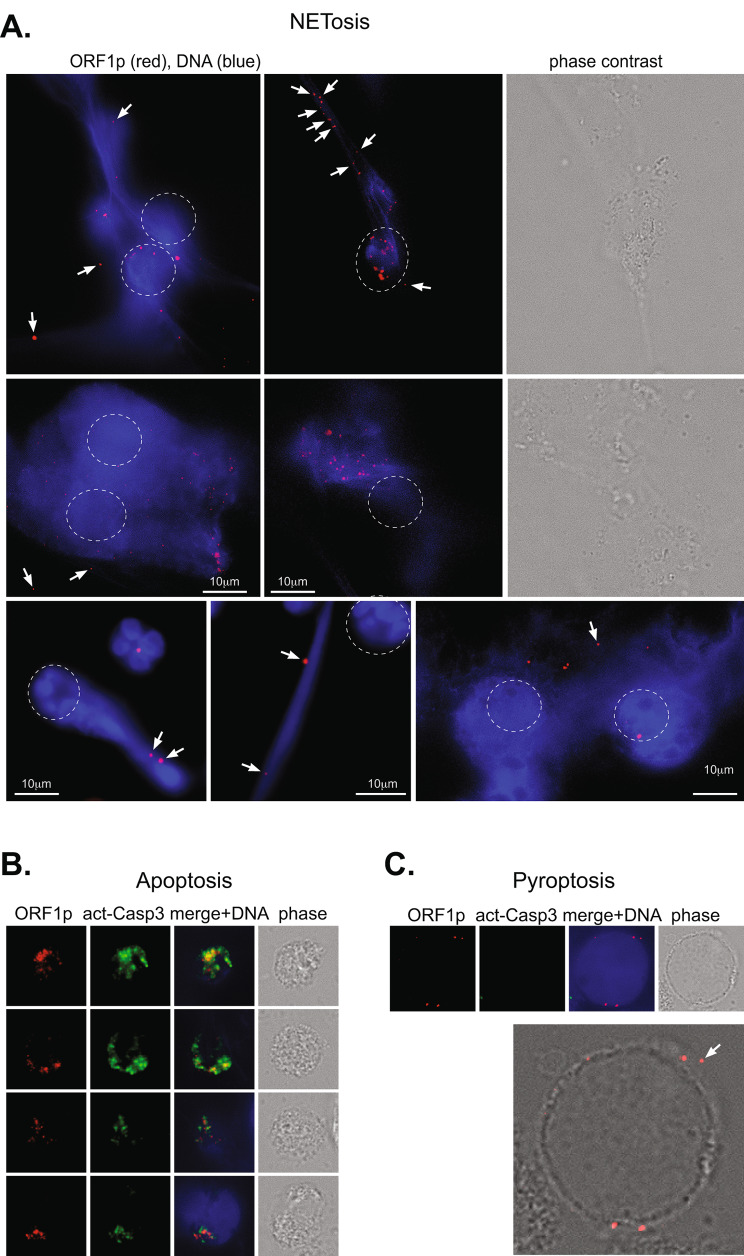
Escape of ORF1p granules from SLE neutrophils during NETosis. **A.**, ORF1p (red) in 6 or 7 SLE neutrophils undergoing NETosis. A normal neutrophil also appears in the bottom left panel. *The approximate location of the neutrophil before NETosis is indicated with a dotted circle.* Externalized, free or DNA-associated ORF1p granules are indicated with white arrows. **B.**, ORF1p (red) remains intracellular in neutrophils undergoing apoptosis as indicated by activated caspase-3 (green). **C.**, ORF1p (red) in a neutrophil undergoing pyroptosis as indicated by cell ballooning and the absence of activated caspase-3 (green). At least one ORF1p granule appears to have escaped (white arrow). The shown images were taken with 100 X magnification

Compared to healthy donor neutrophils, SLE neutrophils are known to more readily extrude part of their nuclear DNA, referred to as neutrophil extracellular traps (NETs), in a process called NETosis [46, 47]. During experiments to visualize ORF1p, two neutrophil spontaneously undergoing NETosis were observed (Fig. 5A, bottom row, left and middle panels). Both contained ORF1p granules that were associated with the DNA (indicated with white arrows in Fig. 5A) well outside of the neutrophil (indicated with a dotted oval). Additional experiments with phorbol ester-induced NETosis confirmed that ORF1p granules dispersed from the neutrophils, often decorating the long extracellular DNA strands or appearing well outside of the cell (Fig. 5A).

For comparison, we examined two other forms of programmed cell death, apoptosis and pyroptosis. The induction of apoptosis by 1 µM staurosporine was verified by the intense intracellular staining for activated caspase-3 (Fig. 5B). The cells noticeably shrank and adopted a more granular appearance. In these neutrophils, ORF1p remained strictly intracellular even after much of the nuclear DNA had been digested. Neutrophils undergoing pyroptosis, on the other hand, inflated into a round balloon-like shape and were devoid of activated caspase-3 (Fig. 5C). Their DNA filled much of the cells in a diffuse manner, while ORF1p granules were located along the plasma membrane, some clearly outside of it (indicated with white arrow).

## Discussion

In this paper, we further strengthen our observation that L1 retrotransposons are expressed in SLE neutrophils [31, 40]. Several of our findings are novel: the more precise quantitation of ORF1p by mass spectrometry in SLE granulocytes and its visualization in cytosolic granules in these cells have not been reported before. The co-localization of ORF1p with the Ro60 and MOV10 proteins, as well as the presence of DNA: RNA heteroduplexes, which depend on ongoing reverse transcription, in SLE neutrophils are also novel findings with implications for the machinery that drives IFN-I production in SLE. Unexpectedly, ORF1p granules and the DNA: RNA heteroduplexes did not co-localize with the DNA-sensor cGAS, but instead with the ZBP1 sensor, which is activated by nucleic acids in the left-handed Z-configuration. The exact nature and origin of the nucleic acids that bind ZBP1 remain to be defined.

Ro60 is a well-known lupus autoantigen [48, 49]. Its normal function is related to the binding and transport of RNAs, such as hY1 RNA [50], and Alu transcripts [51]. The physiological function of MOV10 is in the defense against infection by retroviruses [52] and other RNA viruses [53], such as influenza virus [54], as well as the hepatitis B DNA virus [55]. It is also a potent inhibitor of L1 retrotransposition [56–58]. MOV10 interferes with reverse transcription [55] and acts together with RNASEH2 [59], which degrades the RNA strand of DNA: RNA heteroduplexes. As a helicase, MOV10 may also twist DNA: RNA heteroduplexes into the Z-configuration. This may facilitate recognition by ZBP1, which can signal both to IFNβ production and to the necroptosis programmed cell death program [60–62], which is elevated in SLE [63]. It has also been proposed that necroptosis and NETosis entail the same molecular events [64, 65].

Our findings show that cytoplasmic ORF1p granules can escape neutrophils during NETosis provides a mechanism for subsequent capture of these granules by immune cells for processing and antigen presentation. They may also escape during pyroptosis, perhaps through the gasdermin D pores or after plasma membrane rupture. These granules contain immunogenic ORF1p, as well as other antigenic proteins like Ro60 and MOV10, as well as nucleic acids that can function as adjuvants. Granules consisting of proteins and nucleic acid likely appear virus-like to the immune system and are of the optimal size for potent B cell activation [66]. This model is further supported by the well-recognized status of Ro60 as a key lupus autoantigen [48, 67], as well as the presence of anti-MOV10 autoantibodies in SLE patients [68]. The titers of autoantibodies against ORF1p, Ro60, and MOV10 are positively correlated with each other [68] and they are predominantly seen in patients with an IFN-I gene signature [68]. It has also been reported that anti-Ro60 immunoprecipitates contain L1 transcripts [51] (as well as Alu transcripts); however, since that study converted all precipitated RNA to cDNA for sequencing, it remains unknown if the nucleic acid enriched with Ro60 was RNA, DNA: RNA, or DNA. Our data suggest that DNA: RNA heteroduplexes produced by the ORF2p reverse transcriptase likely were present. These may include reverse transcribed L1 and Alu transcripts.

Because both adult and pediatric SLE patients with active disease have increased autoantibodies against ORF1p compared to patients with inactive disease or healthy adults or children [40], it appears ORF1p granules are predominantly released during flares of the disease and thereby boost an already existing immunity against these granules. This also implies that active lupus is associated with increased expression of L1, as we have shown, and more programmed cell death of neutrophils to release ORF1p granules through NETosis and pyroptosis. Other forms of cell death resulting in membrane rupture may also release these granules.

Our data do not reveal in which anatomical location neutrophil death and the release ORF1p granules occurs. The presence of IgA autoantibodies to ORF1p in children with new-onset SLE is compatible with a mucosal location of ORF1p exposure and immunogenicity. Neutrophils patrol mucosal surfaces and can undergo programmed cell death in this location. Markers of neutrophil death, including elevated levels of S100A8/A9 and circulating DNA in complex with the neutrophil elastase or myeloperoxidase are elevated in pediatric SLE patients with active disease compared to those with inactive disease or healthy children [40]. Hence, activation and NETosis or pyroptosis of ORF1p^+^ neutrophils in mucosal locations may be relevant in early disease. In ongoing disease, released ORF1p granules will likely be decorated by anti-ORF1p, anti-Ro60, and anti-MOV10 autoantibodies further promoting uptake by antigen-presenting cells as well as activation of neutrophils to undergo NETosis, resulting in an escalating positive feedback loop of potential importance in the pathogenesis of SLE.

We further hypothesize that the recently recognized driver role of L1 in cellular senescence, during which the production of IFNβ is part of the senescence-associated secretory phenotype [38], also operates in SLE. Multiple L1 loci are derepressed in senescent fibroblasts, including intact loci that produce catalytically active ORF2p protein, which then generates RNA: DNA hybrids and dsDNA that trigger the DNA sensor cGAS [10] and subsequently IFNβ production [38]. This mechanism also operates in several variants of the Aicardi-Goutières interferonopathy [69], in which reverse transcriptase inhibitors flat-lined the interferon gene signature [70]. Similarly, IFNβ secretion by senescent fibroblasts was abrogated by reverse transcriptase inhibitors in vitro and in vivo [38]. ORF2p-produced cytosolic Alu cDNA also triggered the cGAS-STING pathway in Geographic Atrophy [36, 37]. We have shown that reverse transcriptase inhibitors also reduce IFNβ expression in SLE neutrophils [31]. Our present study suggests that ZBP1, rather than cGAS, may be instrumental in lupus neutrophils (but perhaps not in other cell types) for the intracellular response to L1-catalyzed reverse transcription products, which not only drives IFNβ production, but also promotes neutrophil death via the necroptosis/NETosis pathway, connecting IFNβ secretion with an immune response to ORF1p and associated proteins. The causal connection of these molecular event to SLE disease activity can only be demonstrated by a clinical trial using a L1-selective reverse transcriptase inhibitor.

## Conclusions

Our findings show that neutrophils from lupus patients contain cytosolic granules with ORF1p, Ro60 (a well-known lupus autoantigen), the helicase MOV10 (also a lupus autoantigen), ZBP1, as well as DNA: RNA hybrids, which disappear upon treatment of the cells with an ORF2p-selective reverse transcriptase inhibitor. These macromolecular aggregates have a virus-like composition and size, and they can escape neutrophils dying by the inflammatory programmed cell death pathways of NETosis and (perhaps) pyroptosis, but not when they die by non-inflammatory apoptosis. Extracellular ORF1p granules are likely the form of ORF1p taken up by antigen-presenting cells leading to the prevalent anti-ORF1p autoantibodies in lupus patients. Since the titers of these autoantibodies are higher in patients with active disease, we propose that ORF1p granules are released from dying neutrophils preferentially during disease flares.

## Methods

### Human subjects

Adult SLE patients and healthy controls were recruited through the University of Washington, Division of Rheumatology Biorepository. The study was approved by the University of Washington Institutional Review Board and informed written consent was obtained from all participants according to the Declaration of Helsinki.

### Antibodies and other reagents

The following primary antibodies were used: anti-ORF1p mAb 4H1 (# MABC1152, Millipore, MD), anti-Ro60 (2A4E5, #67149-1-IG, ThermoFisher), anti-MOV10 rabbit polyclonal antibody (# ab80613, Abcam), anti-ZBP1 mAb (clone Zippy-1, AdipoGen # AG-20B-0010), anti-cGAS rabbit mAb (#26416-1-AP, Proteintech), anti-Z-DNA IgG2b mAb Z22 (#Ab00783-3.0, Absolute Antibody), and S9.6, which is specific for DNA: RNA heteroduplexes.

Secondary Antibodies (all from Invitrogen) were: Goat anti-mouse IgG secondary Ab, (AF647 #A-21,235, Goat anti-mouse IgG (H + L) secondary Ab, AF488 #A-11,001, Goat anti-human IgG secondary Ab, AF555 #A21433, Goat anti-Rabbit IgG secondary Ab, AF647 #A-21,245.

In addition, anti-human FcγRII/CD32 Antibody (# MAB1300SP, Fisher Scientific, USA), normal goat serum (ThermoFisher, #50062Z), and Jackson Immuno Research Labs Normal Mouse Serum (# NC9252650, Fisher Scientific) were used to prevent non-specific binding of antibodies. Paraformaldehyde was from ThermoFisher Scientific, Triton X-100 from Millipore-Sigma, Germany, and BlockAid solution from ThermoFisher Scientific.

DNA was stained with 4’,6-diamidino-2-phenylindole (DAPI) or Hoechst 33,342, both from ThermoFisher.

### Reverse transcriptase inhibitor RPT-A

RPT-A is a potent inhibitor of L1 reverse transcriptase [71, 72]. It inhibits the L1 reverse transcriptase enzymatic activity in a biochemical assay with an IC_50_ of 0.03µM, and blocks L1 retrotransposition in a cell-based assay with a bi-directional inducible L1 construct as described [73].

### Neutrophil isolation

Neutrophils were isolated from freshly drawn blood by gradient centrifugation on PolymorphPrep (CosmoBio, USA) according to the manufacturer’s instructions. The cells were washed and suspended in DMEM high glucose (# 11-965-118, Gibco, ThermoFisher Scientific).

### Quantitation of ORF1p by targeted immunoprecipitation-mass spectrometry

Snap-frozen pellets of 20–60 × 10^6^ SLE and control neutrophils were lysed by microtip ultrasonication (1.6 mm diameter probe; 2 Amp; 40 J delivered over 20 s on ice) in 20 mM HEPES pH 7.4, 500 mM NaCl, 1% (v/v) Triton X-100, 5 mM EDTA pH 8.0, and 10x Roche Complete EDTA-free Protease Inhibitors. Crude cell extracts centrifugally clarified (20,000 RCF for 10 min at 4 °C in an Eppendorf 5417R). 95% of the clarified cell extracts were subjected to ⍺-ORF1p immunoprecipitation using 10 µl of affinity medium for 1 h at 4 °C [74]. The medium was washed three times with the same solution used for extraction but containing 2 mM EDTA pH 8.0 and 5x protease inhibitors. Immunoprecipitates were eluted in 2% (w/v) SDS, 40 mM Tris pH 8.0, 2 mM EDTA, and 5x protease inhibitors by incubation at 70° C for 5 min. The eluates were subjected to in-gel tryptic digestion followed by work-up for selected reaction monitoring mass spectrometry (manuscript submitted). For quantitation, the equivalent of 18.75% of the obtained experimental sample and 112.5 amol of isotopically labeled standard ORF1p peptide LSFISEGEIK were analyzed.

### Immunofluorescence microscopy

Freshly isolated neutrophils were cultured for 30 min at 37 °C on 96-well glass bottom plates (Cellvis #P96-1-N) pre-coated with poly-L-lysine (Millipore-Sigma # P4707). The adherent neutrophils were fixed with 4% paraformaldehyde solution for 20 min, followed by two washes with phosphate-buffered saline (PBS). Cells were permeabilized with 0.1% Triton X-100 in PBS for 10 min followed by three washes with PBS. To prevent nonspecific binding, neutrophils were blocked using 10% normal mouse and/or goat serum in BlockAid solution with anti-human FcγRII antibody overnight at 4 °C. Neutrophils were then incubated with a 1:50 dilution of mouse anti-ORF1p mAb 4H1, either unlabeled or directly labeled with fluorophore, followed by three washes with PBS. For indirect immunofluorescence visualization, the cells were subsequently stained with goat anti-mouse IgG secondary Ab conjugated with Alexa Fluor 488 (green) or Alexa Fluor 647 (red). To visualize other proteins, specific antibodies were either directly labeled or stained with a secondary fluorophore-labeled antibody with care not to introduce any cross-reactivity between different primary or secondary antibodies. Negative controls consisted of cells stained without one primary antibody or with only secondary antibodies. Each antigen was also visualized without staining for any other antigens to further ensure that double staining did not create artifacts. Nuclei were stained with either DAPI or Hoechst33342. Images were captured at 40 X, 60 X, or 100 X magnification using a Keyence BZ-X800 Fluorescence microscope and analyzed using BZ-X800 Analyzer software.

### Induction of programmed cell death

To induce distinct forms of programmed cell death, neutrophils adhered to poly-L-lysine-coated glass were treated with 1 µM staurosporine for 4 h to induce apoptosis, 10 µM nigericin for 2–4 h to induce pyroptosis, or 20 nM phorbol ester for 4 h to induce NETosis. Each type of cell death was verified by staining for activated caspase-3 (apoptosis), ASC specks (pyroptosis), or staining for DNA by DAPI or Hoechst 33,342 (NETosis).

### RNA isolation and RNA sequencing

Our procedures for RNA isolation and RNA sequencing were recently published [31].

### Statistical analyses

For non-paired sample sets with non-Gaussian distribution, Mann-Whitney U test was used. GraphPad Prism and IBM SPSS were used for the analyses. All analyses were considered statistically significant at *p* < 0.05.

## Data Availability

The datasets used and/or analyzed during the current study are available for research purposes from the corresponding author upon request. Our RNA seq data are deposited at GEO as GSE261866.

## Abbreviations

BSA: bovine serum albumin
HC: healthy controls
HRP: horse radish peroxidase
IFN-I: type I interferon
LINE-1, L1: long interspersed nuclear element-1
ORF1p: open-reading frame 1-encoded protein
ORF2p: open-reading frame 2-encoded protein
PBS: phosphate-buffered saline
SLE: systemic lupus erythematosus
